# Computer-Aided Detection and Classification of Monkeypox and Chickenpox Lesion in Human Subjects Using Deep Learning Framework

**DOI:** 10.3390/diagnostics13020292

**Published:** 2023-01-12

**Authors:** Dilber Uzun Ozsahin, Mubarak Taiwo Mustapha, Berna Uzun, Basil Duwa, Ilker Ozsahin

**Affiliations:** 1 Department of Medical Diagnostic Imaging, College of Health Science, University of Sharjah, Sharjah 27272, United Arab Emirates; 2 Operational Research Centre in Healthcare, Near East University, TRNC Mersin 10, Nicosia 99138, Turkey; 3 Department of Statistics, Carlos III University of Madrid, 28903 Getafe, Spain; 4 Brain Health Imaging Institute, Department of Radiology, Weill Cornell Medicine, New York, NY 10065, USA

**Keywords:** deep learning algorithm, diagnosis, chickenpox, lesion, outbreak, monkeypox

## Abstract

Monkeypox is a zoonotic viral disease caused by the monkeypox virus. After its recent outbreak, it has become clear that a rapid, accurate, and reliable diagnosis may help reduce the risk of a future outbreak. The presence of skin lesions is one of the most prominent symptoms of the disease. However, this symptom is also peculiar to chickenpox. The resemblance in skin lesions in the human subject may disrupt effective diagnosis and, as a result, lead to misdiagnosis. Such misdiagnosis can lead to the further spread of the disease as it is a communicable disease and can eventually result in an outbreak. As deep learning (DL) algorithms have recently been regarded as a promising technique in medical fields, we have been attempting to integrate a well-trained DL algorithm to assist in the early detection and classification of skin lesions in human subjects. This study used two open-sourced digital skin images for monkeypox and chickenpox. A two-dimensional convolutional neural network (CNN) consisting of four convolutional layers was applied. Afterward, three MaxPooling layers were used after the second, third, and fourth convolutional layers. Finally, we evaluated the performance of our proposed model with state-of-the-art deep-learning models for skin lesions detection. Our proposed CNN model outperformed all DL models with a test accuracy of 99.60%. In addition, a weighted average precision, recall, F1 score of 99.00% was recorded. Subsequently, Alex Net outperformed other pre-trained models with an accuracy of 98.00%. The VGGNet consisting of VGG16 and VGG19 performed least well with an accuracy of 80.00%. Due to the uniqueness of the proposed model and image augmentation techniques applied, the proposed CNN model is generalized and avoids over-fitting. This model would be helpful for the rapid and accurate detection of monkeypox using digital skin images of patients with suspected monkeypox.

## 1. Introduction

The recent multi-continent outbreak of the monkeypox virus presents a severe global health concern due to its rapid spread in 96 countries at the time of writing this manuscript. The world cannot afford another pandemic as the impact of the last one is yet to wind down. One lesson researchers and healthcare practitioners learned throughout the COVID-19 pandemic was the need for accurate and rapid disease detection to prevent future pandemics from leading to unimaginable mortality. As noted during the last pandemic, Artificial Intelligence (AI), with its extraordinary advancement and use in healthcare, has become a powerful tool for disease diagnosis and detection, especially in regions where more sophisticated testing kits are absent. The immense growth and availability of data of healthcare relevance, coupled with improved computational power, have made AI the go-to tool to aid in disease diagnosis, early detection, automation, and treatment [1,2,3]. This significantly impacts general healthcare delivery and assists physicians and other healthcare professionals in their daily activities.

Monkeypox is a communicable Orthopoxvirus that causes a disease with symptoms similar to, but less severe than smallpox [4]. Unlike smallpox, which was eradicated in 1980, monkeypox continues to cause serious global health concerns as it continues to occur in several parts of the world [5]. Monkeypox disease can be transmitted through animal-human and human-human contact [6]. As a result, stopping the spread of monkeypox in a community requires prompt diagnosis, contact tracing, and isolation of those infected. The monkeypox incubation period can be up to 21 days [7]. The febrile stage of illness usually lasts 1 to 3 days with symptoms including fever, intense headache, lymphadenopathy (swelling of the lymph nodes), back pain, myalgia (muscle ache), and severe asthenia (lack of energy) [8]. In recent outbreaks, a case fatality ratio of 1–11% has been reported for monkeypox [9]. Cases are often found near tropical rainforests with disease vectors, including squirrels, Gambian poached rats, dormice, and various monkey species [10]. It can be transmitted through contact with bodily fluids, skin lesions, or internal mucosal surfaces, such as mouth or throat, respiratory droplets, and contaminated objects [4]. Viral Deoxyribonucleic acid (DNA) is the preferred laboratory test for monkeypox to detect viral DNA by polymerase chain reaction (PCR) [11]. However, it is not widely available. Where feasible, the best diagnostic specimens are from the rash–skin, fluid, crusts, or biopsy [4,12,13]. Antigen and antibody detection methods may not be helpful as they do not distinguish between Orthopoxvirus [4] and monkeypox disease. 

Chickenpox, otherwise referred to as varicella, is a severe and highly transmissible disease caused by the herpes virus varicella-zoster virus (VZV). Research has identified only one serotype of VZV, and humans are its only known reservoir. Chickenpox occurs most frequently in people over 50 or those with impaired immune systems [14]. Hence, it is predominant in babies but can also be found in adolescents, adults, pregnant women, and immunocompromised people [15]. Chickenpox manifest as itchy rashes with fluid-like blisters similar to monkeypox, measles, and skin cancer [16]. Over the course of several days, the blisters may pop up and start to leak. Then, they crust and scab over before healing [16]. Chickenpox can be transmitted by breathing in particles from chickenpox blisters or by being in contact with someone who has it [14]. Children in temperate regions are more likely to contract chickenpox than adults, with those in elementary school and younger being most at risk. The normal seasons for the disease are the end of winter and the beginning of spring. Compared to temperate regions, where infections peak in early childhood, adults in tropical regions, notably less populated areas, are more susceptible to these diseases. In tropical settings, the wettest and coolest months are when the highest rates of infection occur [17].

In 1970, a nine-month-old infant in the Democratic Republic of the Congo became the first known victim of human monkeypox in a territory where smallpox had been eradicated in 1968 [18,19]. Since then, most reports have come from the rainforests of the Congo Basin’s rural areas, mainly in the Democratic Republic of the Congo. Reports of human infections have been rising steadily throughout central and west Africa. Monkeypox has been documented in humans in 11 different African nations since 1970, including Benin, Cameroon, the Central African Republic, the Democratic Republic of the Congo, Gabon, Cote d’Ivoire, Liberia, Nigeria, the Republic of the Congo, Sierra Leone, and South Sudan [18]. The fact that the varicella virus, which causes chickenpox, was also detected during an outbreak of monkeypox suggests that there may have been alterations in the transmission dynamics of these two diseases. There have been over 500 suspected cases, and over 200 confirmed cases, with a case-fatality ratio of about 3% in Nigeria since 2017 [18]. Reports of new cases are coming in even now. Monkeypox is an international concern as a disease that spreads beyond west and central Africa. The United States had the first outbreak of monkeypox outside of Africa in 2003, which was traced back to people coming in touch with sick prairie dogs kept as pets. 

**Figure 1:**
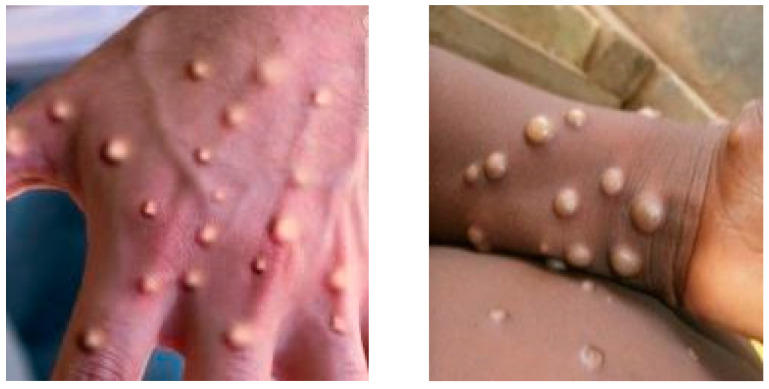
From left: lesion properties of monkeypox and Chickenpox [20,21].

Gambian pouched rats and dormice, introduced to the nation from Ghana, have been living alongside these pets. More than 70 people in the United States contracted monkeypox because of this epidemic. Nigerian tourists have been diagnosed with monkeypox in the following countries: Israel (September 2018), the United Kingdom (September 2018, December 2019, May 2021, and May 2022), Singapore (May 2019), and the United States of America (July 2021, and November 2022). Multiple cases of monkeypox were discovered in various non-endemic nations in May of 2022 [18]. Figure 1 shows the lessons associated with monkeypox and chickenpox.

Due to the similarity of the lesions displayed in the early stages of monkeypox and chickenpox, a clinical diagnosis of monkeypox is difficult. A single misdiagnosis could impact the larger communities as they are both contagious diseases and could spread faster through skin contact and several other routes. They could also result in wrong vaccination and treatment, which can be costly to the government, aid organizations, and infected patients. Even though the mortality rate of the two diseases is relatively low, the spread of the virus could get to immunocompromised patients, which can lead to their deaths. Besides immunocompromised patients, children and older people with low immunity could also get infected with the virus, resulting in death.

Furthermore, healthcare professionals have a knowledge gap due to the rarity of monkeypox before the current pandemic. As a result, there is an urgent need to develop a rapid and accurate way to automate skin lesion detection and classification in human subjects. Among all the advanced deep learning algorithms, CNN is regarded as the most powerful algorithm capable of addressing the problem that our study aims to solve (image classification) [1]. CNN is a subtype of neural network mainly used for image classification [1]. It contains multiple interconnected layers among which is the convolutional layer which helps reduce the high dimensionality of images without losing their information. This makes CNN the best suited deep learning algorithm for this case. This study aims to achieve two things. The first is to train and validate a DL-based model capable of accurately detecting and classifying monkeypox and chickenpox using digital images of human skin lesions. Secondly, we aim to evaluate the performance of the DL-based model with several state-of-the-art pre-trained models. The study’s outcome will be helpful in the early detection and identification of either virus, especially for physicians and aid workers in regions where it is endemic. Ultimately, this will prevent future outbreaks of the disease.

The following points summarize the contributions of this article:

- Proposing a CNN model borrowing knowledge from existing CNN frameworks;
- Training the model with augmented digital skin lesion images of monkeypox and chickenpox;
- Evaluating the performance of the model using unseen and un-augmented digital skin lesion images of monkeypox and chickenpox;
- Finally, evaluating the proposed model performance with the existing state-of-the-art pre-trained model.
- The model will be beneficial for detecting monkeypox and chickenpox lesions, follow-up, and treatment efficacy.

The sections below are organized as follows: Section 2 highlights previous related studies. Section 3 detailed the data and methodology adopted. Section 4 highlights the result obtained and the corresponding discussion. Finally, Section 5 and Section 6 highlight the conclusions and future work, as well as limitations of the study.

## 2. Related Works

No previous study has implemented a DL-based approach to distinctly classify monkeypox and chickenpox, even though there are striking similarities in the lesions of the two diseases. Nonetheless, several studies use a DL-based technique to identify monkeypox and chickenpox.

In a feasibility study by Nafisa Ali et al. [22], several pre-trained deep-learning models were used to detect monkeypox and other similar chickenpox and measles lesions. The dataset was obtained from publicly accessible websites and news portals, and a data augmentation technique was used to increase the total number of datasets. Three commonly used pre-trained DL models were used, including Inception V3, ResNet50, and VGG-16. The result was satisfactory in differentiating monkeypox lesions from other forms of lesions associated with measles and chickenpox. The ResNet50 model recorded the highest performance, with an accuracy of 82.96%. VGG16 and an ensemble of the three models produced an accuracy of 81.48 and 79.26, respectively. In another study by Islam et al. [23], the notion of whether AI can detect monkeypox lesions from digital skin images was investigated. The study introduced the monkeypox skin image dataset in 2022, which is the largest so far [23]. The study implements seven DL models, including ResNet50, DenseNet21, Inception-V3, Squeeze Net, MnasNet-AI, MobileNet-V2, and ShuffleNet-V2-1X. The study concludes that AI has great potential in detecting monkeypox from digital skin images, with a precision rate of 85%. Adler et al. [24] elaborate on the clinical features and management of human monkeypox in a retrospective observational study in the United Kingdom (UK). The study concludes that human monkeypox poses unique challenges in the UK, and even to well-resourced healthcare systems with high-consequence infectious diseases (HCID) networks.

Lee et al. [25] conducted a study on applying a deep-learning model to predict chickenpox. The data used were extracted through web scraping using keywords related to chickenpox. Linear regression and long short-term memory (LSTM) were used to predict chickenpox over time, and the model generated a satisfactory correlation coefficient of 0.97114. However, the linear regression model generates a higher root mean square error of 341.01547. In another study by Alakus & Baykara [26], a DL algorithm was implemented to classify human papilloma virus (HPV) causing monkeypox virus (MPV) and monkeypox DNA sequences. Several DNA mapping methods were evaluated using accuracy, precision, recall, and F1 scores. The result obtained indicates an average accuracy of 96.08% and an F1 score of 99.83%. This further shows that the application of DNA sequences for the classification of warts and monkeypox is feasible, practical, and accurate. Ramadhan and Baykara, [27] implemented an image cropping method and VGG16 model to conduct a binary and multiple classification of coronavirus (COVID-19), normal, and pneumonia. The model produced an accuracy of 97.5% and 99.76% for multiple and binary classifications. 

In a study, Yu et al. [28] presented the application of deep learning technology in the torsional capacity of Evolution of Reinforced Concrete (RC) beams. In the study, a data-driven model based on 2D CNN was designed and fed with imputes. Furthermore, an improved bed swarm algorithm (IBSA) was leveraged to enhance model accuracy and optimize the hyperparameters. The outcome indicates a satisfactory performance in predicting the torsional strength of RC beams. This outcome shows better performance when compared with other ML models.

In another study, Yu et al. [29] proposed a vision-based concrete crack detection technique to diagnose the surface cracks of concrete structures. The goal was to ensure an efficient and time-saving technique that promoted high detection accuracy. The authors used a total of 41,780 image patches of various concrete surfaces for the development and validation of the proposed method. The result indicates the capability of the model to accurately identify crack profiles with wrong predictions of limited areas, demonstrating its potential in practical applications.

## 3. Data and Methodology

Inspired by the effectiveness of AI in detecting Coronavirus disease 2019 (COVID-19), researchers are increasingly considering applying it to the detection of monkeypox and chickenpox lesions using digital skin images of human subjects. However, the lack of monkeypox and chickenpox databases presents obstacles to using AI for monkeypox and chickenpox skin lesion detection.

### 3.1. Data Collection and Description

We collected two datasets for this study: monkeypox and chickenpox. The first dataset is a web-scrapping image collection obtained and used in previous studies [20]. The dataset comprises web-scraping images of monkeypox in one class and images of chickenpox, smallpox, measles, and healthy skin in another class. Only the images in the monkeypox class were used for our study. We double-checked all skin lesion pictures using Google’s reverse image search and other references [23]. Images that were blurry, lacked detail, or were otherwise subpar were eliminated in the first round of screening, leaving only those that were unique to be saved in the second round. From the 228 images obtained [20], 102 are in the monkeypox class, while the other 126 are in the other class (chickenpox and measles). Hence, 102 original images of monkeypox were implemented for this study.

Secondly, the chickenpox dataset was obtained from publicly available case reports through extensive manual searching of the Internet. Automatic web scraping was never used as the sources may be subject to query. The web-scraping images collected fall under commercial and other licenses. For this reason, we provided additional materials for all gathered photographs, such as their universal resource locators (URLs), dates of access, and photo credits (where applicable) [21]. All low-resolution and low-quality images were removed. Also, images with no distinctive label of chickenpox were discarded. The images were resized while preserving their aspect ratio and cropped to the region of interest. Patients in the cropped images of monkeypox and chickenpox have had their eyes covered and any identifying features removed to prevent them from being recognized.

### 3.2. Image Pre-Processing

An image pre-processing step is necessary to enhance an image’s fine details. It removes any unwanted variations to the image and improves the key features [30]. Since all algorithms are vulnerable to noise, properly preprocessed images allow for better segmentation and, subsequently, to better classification. One method of classifying pre-processing methods is the size of the pixel region they target. These techniques function on the neighboring pixels of the sub-image. Images may be improved through enhancement by eliminating distortion and noise. Poor camera quality, a minimal user interface in photography, and environmental conditions can all lead to distorted digital skin images. However, important visual information is sometimes lost in the cases above, making processing too difficult [31]. All of these factors can reduce the contrast in a picture. In this research, we employed image contrast enhancement to better show the details of the region of interest across the two datasets.

Additionally, the original chickenpox and monkeypox dataset underwent an augmentation process utilizing the Python Imaging Library (PIL) version 9.2.0 and the Scikit-image library version 0.19.3. Most state-of-the-art models contain many parameters in the order of millions. To train a model for accurate results, more parameters are needed to learn almost all the features from the data. We need a good amount of data to accommodate all these parameters, and it is standard that DL models often require more data which is only sometimes available. Hence, increasing the number of images and adding some variability to the data is necessary. This technique increased the post-augmentation number of chickenpox and monkeypox images by approximately 44- and 42-fold, resulting in 10,000 images each, as shown in Table 1. However, 50 images each from the monkeypox and chickenpox classes were set aside without augmentation for test data. This will be appropriate for evaluating the performance of the model on unseen skin lesion images of monkeypox and chickenpox. Also, setting aside unseen and un-augmented images for model evaluation prevents knowledge leakage to the model, thereby preventing overfitting.

Image denoising is a process of removing the noise from an image [32]. If not properly addressed, noise from an image will cause a loss of information [32]. This noise often comes from pre-image processing procedures which may include images captured in a low-light situation, sensor illumination levels of a digital camera, faulty memory locations in hardware, and errors in the transmission of data over long distances, etc. Hence, image noise removal is vital and essential to recover the original image from degraded ones. We adopted a deep CNN autoencoder of Denise images present in the dataset, which is trained to reconstruct its input image by learning useful features and representation of the data through an unsupervised learning process. It consists of an encoder and a decoder, both of which are deep neural networks. The encoder networks take in an input image and convert it into a compact representation, known as the latent code, which is then passed to the decoder networks. The decoder network processes the latent code and produces the reconstruction of the input image.

Table 2 shows the augmentation techniques implemented throughout the data augmentation process of the study. Positional and color augmentation techniques were used to achieve several variations of the dataset while maintaining the originality of the data. The range choice is based on our previous experience augmenting data for use. The shear range ensures that the image is distorted along an axis at 20° angles. This help creates or rectifies the perception angles and creates a form of a stretch of the image. With a zoom range at an angle of 20°, the image zooms the image and adds new pixels. Rotation is similar to shear only that it does not stretch the image. Rotation changes the angles of the data that appear in the dataset during training. The zero-phase component analysis (ZCA) whitening is a transformation technique that decorrelates the image pixel. The ZCA preserves the spatial arrangement of the pixels, which is very important when using CNN. Image shift is a geometric transformation that maps the position of every object in the image to the new location of the final output image. By shifting the images, the position of the objects in the image can be changed, giving more variety to the model. This often results in a more generalized model. Flipping allows for the flipping of images in the left-right and up-down directions. All these augmentation techniques improve model prediction accuracy, prevent overfitting, and create variability and flexibility in data used for training [33]. However, there are a few limitations. Augmentation of data requires evaluation systems for quality checks. Also, new research to create new or synthetic data with an advanced application and augmentation techniques like (generative adversarial network) GANs are quite challenging [34].

### 3.3. Hyperparameter Optimization

Because the ultimate goal of an ML researcher, engineer, and data scientist is to achieve optimal performance we building an ML model, it is imperative to optimize hyperparameters. Hyperparameters are variables peculiar to a model whose selection dictate the learning process, determines network structure and can deliver optimal performance of the model. In this study, the hyperparameters of the pre-trained models were left as they are to ensure that knowledge transferred through transfer learning is intact. Any attempt to alter these hyperparameter tuning will lead to a change in the architecture of the model. This may lead to a significant alteration in the model performance. However, the hyperparameters for the pre-trained model were continuously changed until the utmost performance was achieved. The grid search hyperparameter tuning method was implemented to ensure that the hyperparameter with the best result was obtained. Batch sizes of range 10–100 and epochs of range 50–100 were explored. Also, SDG, RMSprop, Adagrad, Adadelta, Adam, Adamax, and Nadam optimizers were explored. Furthermore, a learning rate of 0.001, 0.01, 0.1, 0.2, and 0.3 and momentum of 0.00, 0.2, 0.4, 0.6, 0.8, and 0.9 were explored. Finally, a batch size of 32, 50 epoch, SDG optimizer, the learning rate of 0.01, and momentum of 0.00 were selected as they achieved optimal performance.

### 3.4. Proposed Model

The deep learning framework adopted for our proposed model was designed to illustrate the capability of a CNN model developed from scratch with unique modification and peculiarity to the data in use to attain optimal performance. The proposed model conforms with the data used and the frame of the problem we intend to solve. We designed the DL network as a simple CNN model and improve it through the addition of layers (convolution, pooling, and dense) and hyperparameter tuning until the utmost performance was achieved. While tuning the hyperparameter, we kept in mind avoiding overfitting and underfitting to ensure adequate generalization of the unseen data. This is visible in the performance of the model when compared with the performance of the state-of-the-art pre-trained model evaluated in this study.

**Figure 2:**
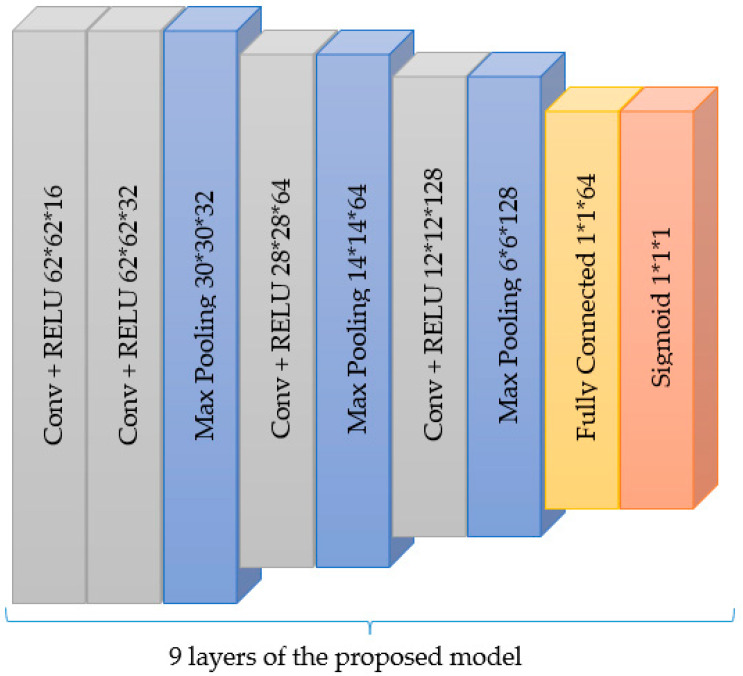
The basic architecture of the proposed model.

We adopted a convolutional neural network (CNN) architecture using the detection method of classification, which determines the output information from a single image. The maximal diameter of the region of interest in the image is of great clinical importance. Our CNN architecture consists of a 2-dimensional (2-D) CNN architecture. The network comprises four convolutional and three max-pooling layers applied after the second and fourth convolutional layers, as shown in Figure 2. The two layers used kernel sizes of 3 × 3 and 2 × 2, respectively. A series of 2 fully connected layers with 64 and 2 units provided high-level reasoning before the final sigmoid classifier layer. Details regarding training are as follows: Adam, a gradient-based stochastic optimizer, was utilized with a batch size of 32 and a dropout of 25% on the convolutional and fully connected layers, respectively. We used the binary cross entropy loss to compare the predicted probabilities to the actual class output, which can either be 0 or 1. Finally, we compile the model using accuracy metrics. The rectified linear activation function (ReLu) was the activation function of choice across the entire network before the final sigmoid activation function. In training the CNN using our dataset, we used a part of the training set as a validation dataset and tested the model accuracy on the unseen test dataset. Training with too many or too few epochs may lead to overfitting or underfitting a DL model. As a result, we implemented the early stopping method. This method allows for a specific arbitrary number of training epochs to be assigned and stopped once there is no improvement in model performance. Also, we adopted the dropout regularization technique after the third max pooling layer and in the dense layer. Dropout regularization is an easy-to-use regularization technique. It produces a simple and efficient neural network by turning off some neurons during training. Simple neural network results in less complexity and, in return, reduce overfitting.

## 4. Results and Discussion

All techniques and procedures were implemented using the Keras package and the Python programming language, including data cleaning, image pre-processing and augmentation, model building, model training, and evaluation.

**Figure 3:**
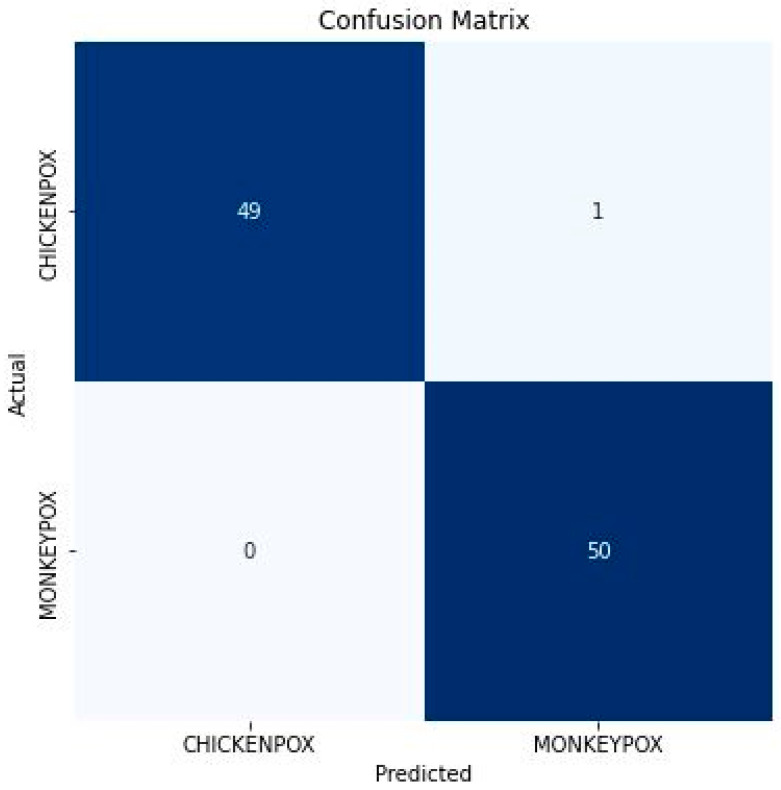
Confusion Matrix of the proposed model.

After model training, hyperparameter tuning, and evaluation, the result shows the model’s capability to significantly perform satisfactorily and classify monkeypox and chickenpox skin lesions without overfitting or underfitting problems. When evaluated on unseen test datasets, the proposed model generates an accuracy and loss of 99.00% and 0.15163. This demonstrates that when provided with unseen images of monkeypox and chickenpox, the model can classify them. The test accuracy is an important metric that depicts the fraction of predictions the proposed model got right. Also, of the 100 test images evaluated, the model correctly identifies 50 as monkeypox (True Negative) and 49 as chickenpox (True Positive), as shown in Figure 3. True Negative (TN) tells how often a model correctly classifies monkeypox as monkeypox. Likewise, True positive (TP) tells how many times a model correctly classifies chickenpox images as chickenpox. This means that 99 images of chickenpox and monkeypox were accurately categorized.

Nonetheless, 1 image of chickenpox was incorrectly classified as monkeypox (False Positive (FP)), while no image of monkeypox was incorrectly classified as chickenpox (False Negative (FN)). False positive tells how many times a model incorrectly classifies chickenpox as monkeypox. In contrast, false negative tells how many times a model incorrectly classifies monkeypox and chickenpox. This further clarifies that the model performs incredibly well and may be combined with other known symptoms for the final diagnosis of the disease.

Accuracy alone does not give the full picture of model performance. To fully evaluate the performance of a model, the precision, recall, and F1-Score must be examined. Unfortunately, precision and recall are often in tension. As shown in Table 3, a mean precision of 99.00% produced by our proposed model indicates the quality of positive prediction made by the proposed model. A corresponding mean recall of 99.00% shows the ratio of monkeypox images correctly classified as monkeypox to the total number of monkeypox images. The higher the recall, the more monkeypox images detected and vice-versa. The F1 score defines the harmonic mean of precision and recall. It does this by combining the precision and recall of a classifier into a single metric by taking the harmonic mean. With a mean F1 score of 99.00%, the prosed model generates a satisfactory harmonic mean, precision, and recall.

**Figure 4:**
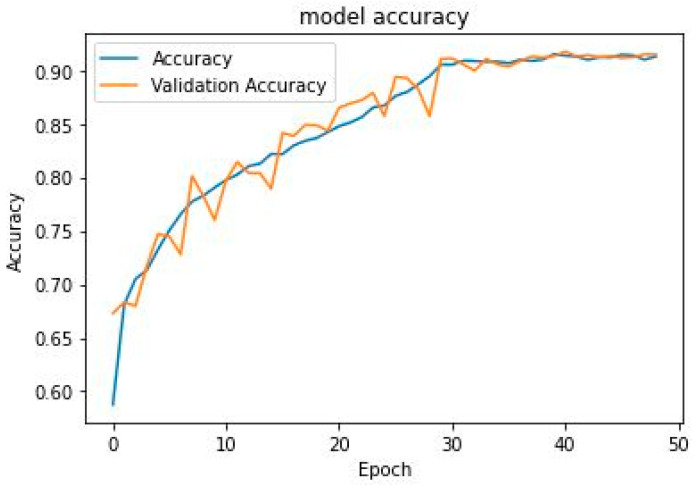
The learning curve for training and validation accuracy.

**Figure 5:**
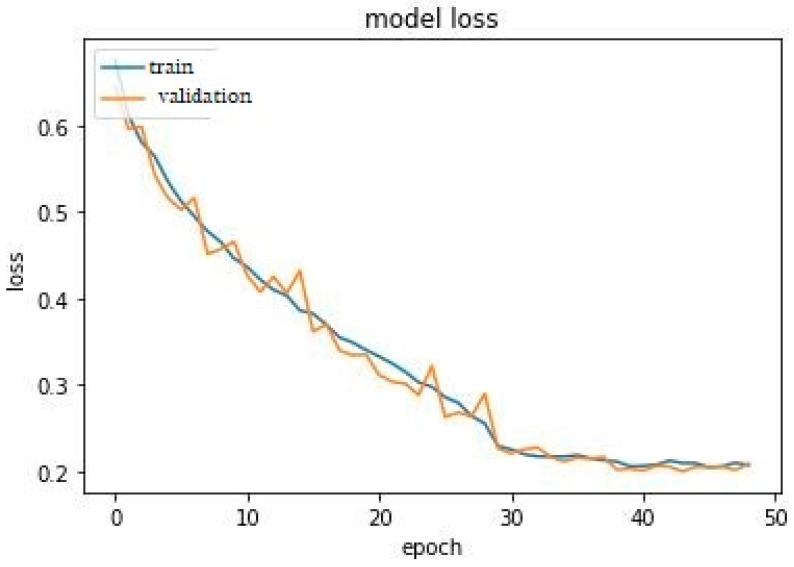
The learning curve for training and validation loss.

Learning curves are important metrics in DL to optimize internal parameters [35]. It is a plot of the model learning performance over time or experience. Reviewing the learning curves of a model during training can be used to diagnose problems with learning, such as an overfit or underfit model, and the training and validation datasets are suitably representative. Figure 4 and Figure 5 show the proposed model’s accuracy and loss learning curve. The proposed model generated a good fit from about the 30th epoch and sustained the excellent fit to about the 48th epoch for both the training and validation accuracy. Consequently, a good fit was generated from the 28th to the 48th epoch for both training and validation loss. The two learning curves indicate that our proposed model neither overfits nor underfit the data. Hence, the proposed model is capable of generalization on unseen test datasets.

Furthermore, we compared the performance of the proposed model with the state-of-art pre-trained model, including VGG16, VGG19, ResNet50, AlexNet, and InceptionV3. This comparison was made to examine the feasibility of the pre-trained model to successfully classify digital images of monkeypox and chickenpox lesions in human subjects. These models have, in previous literature, demonstrated excellent classification performance [36,37,38,39,40]. The VGG16 consists of 3 × 3 convolutional filters using Factorized Convolution to enable feature extraction while ensuring that overfitting of training data is avoided. The VGG19 is a modified version of the VGG16 with 19 convolutional layers. The ResNet50 model employs residual nodules where convolution operations are followed by Batch Normalization and ReLu non-linearity and is based on residual learning [41]. These building blocks expedite input propagation and improve feature extraction. The InceptionV3 is a CNN for assisting in image analysis and object detection. It focuses on burning less computational power by modifying the previous Inception architectures. With the ‘stacked’ Inception nodules, the InceptionV3 performs better despite having fewer parameters than VGG16. The AlexNet CNN, which contains eight layers, 5 of which are convolutional layers and three fully connected layers, shows that the task of image classification can be tackled using deep CNN.

We could determine how many more layers were necessary for optimal performance by utilizing transfer learning on the training data. In the best-case scenario, the last classification layer must be adjusted, while the rest must remain unchanged. Weight initialization is vital in designing a neural network model. It typically involves adopting types of activation functions, the number of inputs to the nodes, etc. The weight initialization for all pre-trained models implemented for this study adopt transfer learning. That way, knowledge gained from a previously solved problem is used for a second related problem. For the 5 pre-trained models used, we froze the layer trained on the large image dataset and modified only the last layer to align with the study’s classification goal. By freezing the layers, the weight of the layer will not be updated. This further indicates that the feature extraction later frozen will not be trainable. This, higher accuracy can be achieved for smaller datasets.

Our proposed model results significantly better than state-of-the-art pre-trained models, as seen in Table 4. With an accuracy, TP, and TN of 99.00%, 49, and 50 respectively, the proposed model outperformed the closest-performing model AlexNet. Our model wrongly classified only 1 (1%) image. That is significantly better than the 2 (2%) images misclassified by the AlexNet model. Furthermore, VGG16 and VGG19 perform the worse with an accuracy of 80.00%, respectively, and misclassify 20 (20%) of the total test images. Training a model on millions of non-relatable types of datasets and transferring the knowledge to a different dataset may not always be the best approach, as the knowledge transferred may not be of utmost usefulness for the new dataset. Also, applying a one-model-solve-all-problem approach cannot be a reliable means of solving new and unique problems, as classification problems are specific and unique. Furthermore, medical datasets are growing, and so are their features. Hence, adequately designed proposed CNN models can outperform state-of-art pre-trained models.

Finally, all models’ performance was evaluated using exactly the same training, validation, and test sets. This means all models were trained and tested using the same training and test dataset. The proposed model generates an accuracy and loss of 95.00% and 0.21792. This is higher than the state-of-the-art models (AlexNet and inceptionV3), with the most superior performance of 91.00% accuracy. Furthermore, the VGG16, ResNet50, and VGG19 models obtained test accuracies of 90.00%, 84.00%, and 77.00%, as shown in Table 5. This further indicates the use of DL frameworks for the classification of monkeypox and chickenpox.

## 5. Conclusions and Future Work

The early detection of monkeypox and chickenpox is vital for the rapid and adequate treatment of the disease. This ultimately prevents outbreaks and mortality associated with the disease. The similarity in the lessons of monkeypox and chickenpox can create a problem of misdiagnosis, especially in endemic regions where communicable disease experts are insufficient.

The outcome of this study highlights the possibility of accurately classifying commonly characterized skin lesions associated with monkeypox and chickenpox using a DL framework. With the current monkeypox outbreak, a DL approach can be implemented independently or with communicable disease experts in regions where the disease is endemic. This would be useful in the rapid detection of the disease. Hence, preventing preventable monkeypox and chickenpox outbreak in the future. 

Future work can be carried out on developing a CNN model capable of accurately classifying monkeypox, chickenpox, and skin cancer lesion, as there are similarities in the skin lesion peculiar to the three diseases. Also, another approach could be to use deep learning to analyze text data such as medical reports or clinical notes and classify them as either related to monkeypox or not related to monkeypox. This could involve training a long short-term memory (LSTM) network or some other type of natural language processing (NLP) model on a dataset of labeled text data.

## 6. Limitations

However, the study has some limitations. Data availability is a great challenge as no large dataset is available. This makes using data authentication techniques such as annotation and usage nearly impossible. Also, the rare occurrence of monkeypox and chickenpox makes the disease less studied. Thereby limiting knowledge of the disease among physicians. Ultimately, this could lead to the disease not being appropriately diagnosed, leading to the spread of the disease and eventual outbreak. 

## Figures and Tables

**Table 1:** The distribution of the dataset.

Reference	Data Class	The Original Number of Images	The Number of Data Augmented	The Number of Images after Augmentation	The Number of Unaugmented Images Reserved as Test Dataset (for Evaluation)
[20]	Monkeypox	102	52	10,000	50
[21]	Chickenpox	240	190	10,000	50

**Table 2:** Image Augmentation settings.

S/N	Augmentation Settings	Range
1	Shear range	0.2
2	Zoom range	0.2
3	Rotation range	0.2
4	ZCA whitening	False
5	Width shift range	0.3
6	Height shift range	0.3
7	Channel shift range	0.2
8	Vertical flip	True
9	Horizontal flip	True

**Table 3:** Performance evaluation metrics of the proposed model.

	Precision	Recall	F1-Score	Accuracy
Chickenpox	100.00%	98.00%	99.00%	99.00%
Monkeypox	98.00%	100.00%	99.00%	
Weighted average	99.00%	99.00%	99.00%	

**Table 4:** Proposed Model versus state-of-art pre-trained models.

Network	Class	Precision %	Recall %	F1 Score %	TP	FP	FN	TN	Accuracy %
Proposed Model	Chickenpox	100.00	98.00	99.00	49	1	0	50	99.00
	Monkeypox	98.00	100.00	99.00					
VGG16	Chickenpox	81.00	78.00	80.00	39	11	9	41	80.00
	Monkeypox	79.00	82.00	80.00					
VGG19	Chickenpox	86.00	72.00	78.00	36	14	6	44	80.00
	Monkeypox	76.00	88.00	81.00					
ResNet50	Chickenpox	76.00	94.00	84.00	47	3	15	35	82.00
	Monkeypox	92.00	70.00	80.00					
AlexNet	Chickenpox	98.00	98.00	98.00	49	1	1	49	98.00
	Monkeypox	98.00	98.00	98.00					
InceptionV3	Chickenpox	88.00	90.00	89.00	45	5	6	44	89.00
	Monkeypox	90.00	88.00	89.00					

**Table 5:** Model evaluation using the same training and test dataset.

	Test Accuracy %	Loss %
Proposed Model	95.00	0.21792
AlexNet	91.00	0.49988
InceptionV3	91.00	0.17745
VGG16	90.00	0.22484
ResNet50	84.00	0.55174
VGG19	77.00	0.44056

## Data Availability

The data used for this study will be provided upon request.
